# Challenges in AI Based Tumor Board Case Summarization and Recommendations

**DOI:** 10.21203/rs.3.rs-9916397/v1

**Published:** 2026-06-16

**Authors:** Wen-wai Yim, Hendrik Damm, Tabea M. G. Pakull, Sam Preston, Timothy Keyes, Timothy John Ellis-Caleo, Zhaoyi Sun, Meliha Yetisgen, Noel Codella, Mu Wei, Faraah Bekheet, Joel W Neal, Nigam Shah, Bahadır Eryılmaz, Felix Nensa, Elisabeth Livingstone, Christoph M. Friedrich, Georg Lodde

**Affiliations:** 1 Microsoft Health AI, USA; 2 Department of Computer Science, University of Applied Sciences and Arts Dortmund (FHDO), Germany; 3 Institute for Medical Informatics, Biometry and Epidemiology (IMIBE), University Hospital Essen, Germany; 4 Institute for Transfusion Medicine, University Hospital Essen, Germany; 5 Department of Medicine, Stanford University, USA; 6 Department of Biomedical Informatics and Medical Education, University of Washington, USA; 7 Department of Dermatology, University Hospital Essen, Germany; 8 Institute for Artificial Intelligence in Medicine (IKIM), University Hospital Essen, Germany; 9 Institute of Diagnostic and Interventional Radiology and Neuroradiology, University Hospital Essen, Germany

## Abstract

Tumor boards, recurring meetings at hospital institutions, assemble multiple cancer specialties (e.g. medical oncology, pathology) to discuss oncological care for patients. In this work, we formally describe the tasks of tumor board case summarization, options generation, and meeting outcomes prediction as AI problems. We study datasets from 4+ medical institutions, providing the performance of available state-of-the-art large language models (LLMs) across DeepSeek, GPT, Gemini, Qwen families, with human-expert evaluations. In total, medical oncologists created reference texts and human judgments across tasks totaling ~10k and ~13k respectively. Results revealed clinicians rated LLM-generated case summaries 3.57–4.59 out of 5.0 across institutions, but struggled with the recommendations generation task, averaging 2.0–3.6. Clinical alignment studies revealed modest correlations of 0.2 for case summarization subtasks, but higher for recommendations at 0.6 for LLM-as-Judge metrics. Our proposed TBFact metrics were shown to be competitive with LLM-as-Judge metrics in longer free text settings, suggesting a promising direction towards explainable metrics. This work, the largest study with expert grading with both granular and direct assessments on multiple axes (e.g. completeness, factual accuracy), revealed surprising nonlinear relationships between granular criterion-level scores and overall direct assessments. Finally, our study revealed experts frequently assigned high ratings to responses that differed substantially from their own reference texts, challenging the core assumptions underlying reference-based automatic evaluation metrics.

## 1 Introduction

Oncological case management regularly requires collaboration from multiple departments, including the pharmacy, cancer infusion clinics, and the radiology imaging department, to provide adequate surveillance and control for multiple side effects and systemic treatments. When active intervention is taking place, other departments such as surgery and radiation oncology become involved for consultation and treatment. If specialty testing is required, pathology or even outside genetic testing provide additional support. In the event of a newly discovered disease, new disease progression, new symptoms, or requirement for treatment changes, it is necessary to involve one or multiple of these specialties to explore the feasibility of next steps, decide on optimal testing direction (weighed against disease risk, patient comfort, and efficacy), or to choose between alternative treatments with varying trade-offs. Tumor board meetings are regular meetings in which specialists, particularly from medical oncology, radiation oncology, and surgery, discuss patient cases to support collaborative clinical decision making.

**Figure 1.**
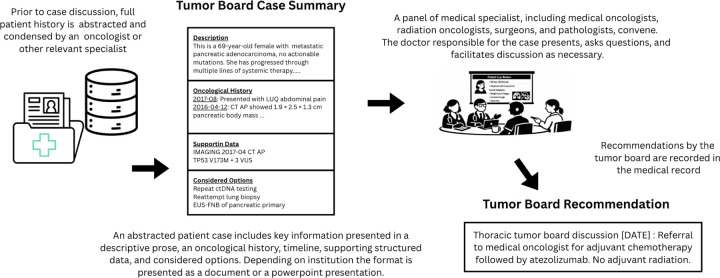
Tumor Board Case Summary and Recommendation Task Description

For effective meetings, prior to each tumor board meeting, each patient’s case history must be organized to present concise and relevant facts to the panel of domain experts. Pertinent facts include patient disease progression, past treatments, and current predicament. Additional deliberation points may include possible alternative treatments, including clinical trials, usually geographically local (e.g. patients may realistically only consider clinical trials within 50 miles driving distance). The presenting physician, who is currently responsible for the patient’s care, may be a medical oncologist, but can also be a surgeon or radiation oncologist when the patient is actively under their care. Depending on the personal record-keeping practices, institutional workflows, and cancer type, preparation of a tumor board case summary may be as short as 5 minutes or as long as 20 minutes to an hour or more, per case. The duration of case presentations during tumor board meetings also varies widely. In some specialties, a case may be discussed in five minutes, while in others discussions may last twenty minutes or longer. With multiple patient cases to prepare, physicians may spend several hours organizing presentations for tumor board meetings. To reduce this physician burden, institutions often employ medical assistants to support portions of the preparation process. After the meeting, the physician communicates the board’s recommendations to the patient and may document the discussion and decisions in the clinical chart. Figure 1 illustrates the clinical workflow in practice.

Prior work related to tumor boards in scientific literature can be grouped into two categories: (a) tumor board operational efficiency and efficacy and (b) testing LLMs for tumor board treatment recommendation prediction. For the former, the focus topics include on optimizing tumor board structure and efficiency^1^, identifying benefits and outcomes^2^, studying tumor board program impacts^3^ and measuring adherence^4^. For the latter, typical works require human-created vignettes (a hand-written patient history) or patient summary descriptions as inputs, calls to commercial LLMs like ChatGPT-3.5, 5o or Claude 3, then a human evaluation of the output through a Likert rating. Typically these previous works involve single institutions with 1–2 evaluated systems. For example, a series of studies by Schmidl et al evaluate head and neck cancers for GPT4o and GPT4, Claude3 and GPT4 and ChatGPT-3.5 and GPT4^5–7^, with 100, 50, and 20 cases, respectively, with often high ratings of 4.7/5.0 for newer models. In Sorin et al.^8^, the authors explore ChatGPT-3.5’s ability to summarize as well as provide tumor board recommendations and explanations for 10 breast cancer cases. The study reported an average summarization score of 4 out of 5 and an 80% agreement rate with gold standard treatment recommendations. In Griewing et al.^9^, 20 fictitiously created cancer cases were used to challenge ChatGPT-3.5 in the concordance of treatment categories against gold standard^1^. Results for this categorical classification was at 58.8%. In Benary et al^10^ the authors used 10 fictitious cases with several models: ChatGPT, Galactica, Perplexity, and BioMedLM. Presenting outputs, they took physician ratings on likelihood of generated by AI as usefulness in the content. It was found that doctors were very likely to discern AI generated content and overall clinical usefulness ratings were low. Finally, Aghamaliyev et al.^11^ further evaluated the accuracy of ChatGPT 3.5 in generating exact treatment recommendations for 115 gastrointestinal cancer cases, including specific chemotherapy regimens and drug names. The authors found that concordance, at 83% for broad categories, would drop to 65% when measured against exact chemotherapy recommendations. Table 1 summarizes prior work on treatment recommendation generation using real hospital data. Extending beyond previous studies, we evaluate additional previously unstudied cancer types across multiple institutions and compare a broader range of LLMs from several model families, including DeepSeek, GPT, Gemini, and Qwen, realistically available per institutions.

One prior work, Blondeel et al.^12^, describe their use of a GPT-4.1 agent to generate a tumor board case summarization. The authors further describe a new metric with analysis of intermediate entailment-based classification performance. We extend this work by formalizing the multifaceted aspects of the case summarization problem. Specifically, we divide the free text and semi-structured sections, as well as partition option prediction as a separate task. We provide domain-motivated concrete definitions of oncological facts, empirically measure performance of fact classification for the TBFact metric, using LLMs compared with human domain experts across multiple model families, and provide in-depth descriptions of their challenges. We introduce a new reference-free metric TBFact-RF and study its efficacy. Finally, unlike previous work, we provide multiple medical oncologists, comparison of summaries, and test end-to-end multiple correlations with human experts across multiple automatic natural language generation (NLG) evaluation metrics.

Our contributions include the following: To our knowledge, this is (a) the first work to comprehensively benchmark the tumor board case summarization and recommendation AI tasks across 4+ datasets/institutions and specialties for 4 LLM families. It is (b) the largest work with human medical oncologist judgments across four axes (completeness, factual accuracy, relevance/helpfulness, overall), including a study of human granular rubric grading. We provide (c) a large-scale study of aligning automatic metrics with human preferences, uniquely examining a rare multi-reference setting with paired individual human assessments and fine-grained rubric-based evaluation. We (d) propose and study TBFact and TBFact-RF (reference-free) a domain-motivated fact-based evaluation metric.

## 2 Task Description

### 2.1 Case Summarization and options generation

In preparation for a tumor board meeting discussion, case summaries and next step options are collected ahead of time. Depending on the institution, the prepared materials are organized into PowerPoint slides, structured reports, or personal notes. Presentation follows the format of the case summary document – either presented with slides or verbally read aloud during the meeting. Next step options or questions to the panel are optional elements prepared by the presenting physician.

To capture common aspects across tumor board presentations, in collaboration with medical oncologist experts from multiple institutions, we defined a generalizable set of generation tasks with four sections:

- **Description:** A prose summary description providing patient history background, including but not limited to age, demographics, stage, and comorbidities. It may include a question to be addressed in the tumor board meeting and also patient preferences.
- **Oncological History:** A semi-structured timeline of the patient’s significant oncological events. These may include past treatments, critical testing results, or changes in patient’s overall health.
- **Supporting Data:** Structured data or additional excerpts of medical record material useful for review at a tumor board. These may include excerpts of imaging reports, actual images such as pathology or imaging scans, or graphical displays of lab values. This is represented as a list of dates and text excerpts.
- **Considered Options:** A list of potential next step options for the patient’s case at the current time point, with a brief description of the option’s rationale. If a treatment or clinical trial, it may include links to academic publications or trial pages. This is represented as short descriptions, rationales, and links.

**Figure 2.**
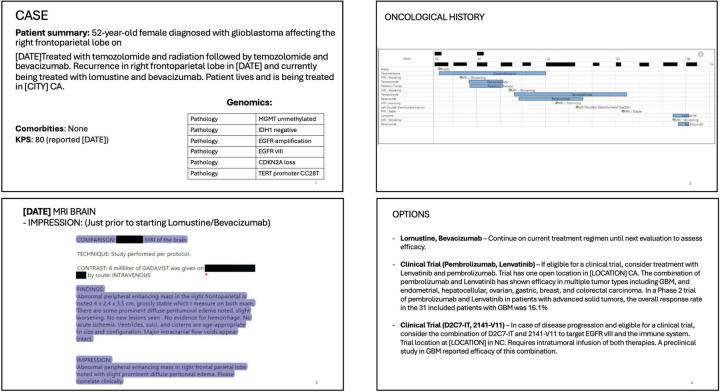
Tumor Board Case Presentation Examples. (TOP-LEFT Description, TOP-RIGHT Oncological History, BOTTOM-LEFT Supporting Data, BOTTOM-RIGHT Considered Options)

Figure 2 presents an example of a tumor board presentation in PowerPoint form. To gather insight on the variations of tumor board case reporting, we conducted a survey of 15 US and UK based doctors to understand typical case summary formats and durations. In our findings, 8/15 doctors reported document-based templated tumor board textual reports, 3/15 used power points (including images, graphs, etc.), and 1/15 other (e.g., reading personal notes out loud). For actual case discussions during tumor board meetings, 2/15 reported spending less than 10 minutes, 9/15 10–20 minutes, 3/15 20–30 minutes, 1/15 30 minutes or more. Eight out of 15 reported using bulleted brief summaries, 6/15 another concise paragraph, 1/15 other. General consensus among experts was that preparing presentations was time-consuming. All reported experiencing variations when practicing at different institutions, as well as patient case complexity being another major factor for content length differences. For simplicity we refer to the first three subtasks as the Case Summarization task and the generation of considered options as the options generation task.

### 2.2 Tumor Board Recommendations Prediction

Building on prior work, the objective of this task is to predict the next steps for the patient. Motivated by naturally occurring data in patient records, which only records outcomes of tumor board meetings, this task captures the final recommendations of these meetings. Depending on author preference and case complexity, these final recommendations may be very short and concise (e.g.”She was presented at the tumor board today. She has been recommended neoadjuvant chemoimmunotherapy.”) versus detailed and multi-factored (e.g. “Proceed with palliative external beam radiation to the left inguinal mass and right iliacus (36 Gy in 12 fractions) and consider a targeted boost to the pancreatic head if symptomatic. After radiation, initiate salvage BR + polatuzumab if tolerable. Continue pain control, nutrition support, and refer to palliative-care services”); they may include general next test recommendations or fairly detailed treatment regimens. Unlike prior work, this task extends beyond a simple categorical classification of next step treatments such as radiation, systemic, or surgery; with the input for this task as the entire patient record (instead of a human-authored vignette). Further examples can be found in Supplementary Materials.

### 2.3 Automatic Evaluation Correlation with Human Experts

Human expert judgments are laborious, time-consuming, and expensive. Meanwhile, although automatic metrics are cheap and efficient, they do not always capture all important aspects of clinically relevant quality. In order to understand how well current automatic metrics correlate with human expert behavior, we experiment with multiple evaluation metrics and compare them to human judgments. We measure clinical alignment by calculating Kendall’s tau, Pearson, and Spearman coefficients.

We study common evaluation metrics: BLEU^13^ and ROUGE^14^. As well, we test LLM-as-judge and our newly proposed TBFact scores. The configurations for the mentioned metrics are available in our CODE. Human evaluations, described in the methods section, include 1–5 Likert style ratings of case summarization and TB recommendations prediction. For the case summary description generation, we additionally collected detailed human rubric evaluations for two datasets (DS1 and DS3) which provides further insight into granular scoring.

## 3 Datasets

In this work, we studied five datasets drawn from two in-house, proprietary sources and three academic medical centers. The two in-house, proprietary datasets comprised cohorts from a remotely conducted molecular tumor board that covered multiple cancer types (DS1) and from a regional United States cancer hospital (DS2). The three academic medical centers were Stanford University (DS3), University Hospital Essen (DS4), and University of Washington (DS5). Across these datasets we defined five inter-related tasks: tumor board case summarization, case options generation, tumor board recommendations prediction, tumor board summary rubric evaluation, and tumor board summary overall evaluation. Table 2 reports the number of cases and the task types performed for each dataset.

Table 3 provides summary data statistics per task. The counts for non-evaluation tasks represent the number of gold standards available for the dataset-task. A subset of cases received annotations from more than one expert, so the gold-standard counts exceed the case counts in Table 2. For the evaluation tasks, the number of judgment instances is the product of the number of gold-standard items rated, the number of baseline systems (e.g. GPT, Qwen), the number of judgment axes, and the number of summary sections. For example, 10 cases rated by 3 baseline systems (GPT, Gemini, Qwen) on 2 judgment axes (completeness, overall) for a single section yield 10×3×2 = 60 judgments.

## 4 Methods

### 4.1 Cohort Identification

#### DS1: Proprietary Molecular Tumor Board Dataset

This proprietary dataset is comprised of United States molecular tumor board discussions, conducted remotely from 2019–2023, and accompanying exports of clinical notes of various types relevant to oncological care (radiology, biomarker reports, outpatient clinic reports, etc.). Discussions from the tumor board meetings included the case summary, though final recommendations were not saved. Cancer types were mixed including a variety of cancers (e.g. gliobastomas, adenocarcinomas, etc.). Hospital institutions from across the United States contributed to this collection. Patient clinical note history was prepared by taking all notes prior to the tumor board summary date. To fit within the context, we experimented and identified a cut-off of 30 most recent clinical notes. This cut-off was deemed sufficient after studying the notes, which were already filtered for relevance to the tumor board as part of the dataset. Clinical notes in this dataset were large exported documents that may include multiple concatenated documents.

#### DS2: Proprietary Regional Cancer Center Dataset

This dataset comprised of clinical notes of various oncological specialties (e.g., radiology, biomarker, surgery) from a single regional cancer center from 2014–2025, with ICD diagnosis codes C34 for lung cancer and C50, D05 for breast lung cancers. To identify tumor board meeting dates, all notes were checked against a regular expression with “tumor board” mentions. Afterwards, medical annotators read the notes and identified several the exact tumor board date and the tumor board recommendation. For completeness, we only kept patient case - tumor board date combinations with explicit recommendations and named tumor board dates. To identify input patient history records, similar to DS1, we used a cut-off of 30 most recent clinical notes. This dataset, already pre-filtered for notes associated with cancer care, primarily consisted of outpatient notes.

#### DS3: Stanford University

Data included 50 cases from the Thoracic Tumor board at Stanford Health Care in 2025, which were examined as part of an effort to improve the quality of the tumor board (approval #82057). Dates and patient identifiers were manually stored from actual meetings. Subsequently, paired patient histories were constructed by retrieving the notes from those patients within a 180 day window, achieved using a GPT with FHIR access.

#### DS4: University of Essen

Cases were identified by querying a FHIR-based clinical data repository at the University Hospital Essen. A SQL query retrieved all DiagnosticReport resources categorized as tumor board protocol associated with the dermatology department, restricted to reports issued on or after January 1, 2024. The resulting protocols were downloaded in plain-text format and further filtered to retain only documents explicitly titled as interdisciplinary or internal skin tumor board protocols. Duplicate presentations of the same patient were removed using the patient ID. Final manual evaluation samples were drawn from ICD C43 (malignant melanoma of the skin) cases.

For each identified tumor board case, longitudinal clinical progress notes (excluding inpatient nursing documentation) were retrieved from the FHIR server. Only notes dated strictly before the tumor board report date were retrieved. Additionally, patient history entries matching tumor board recommendation patterns and sharing the same date as the diagnostic report were removed to prevent target leakage. The remaining notes were retained as the patient history input for each case.

#### DS5: University of Washington

Tumor board cases were identified from a cohort of diffuse large B-cell lymphoma (DLBCL) and prostate cancer patients, from the Fred Hutch Cancer Center from 2015 to 2018. Similar to DS2, tumor board meetings were identified by first identifying “tumor board” mentions from clinical notes. Qwen3-14B was used to extract tumor board date and final recommendation. Afterwards, manual review and correction was conducted to ensure tumor board date and recommendation accuracy.

Patient history per patient-tumor board meeting date was constructed from longitudinal clinical notes documented before the tumor board date. To focus on information relevant to oncology decision-making, we filtered notes by note type and provider specialty. Included note types covered major clinically relevant categories, such as oncology and radiation care, imaging, pathology, and molecular testing, surgery and procedures, consultations, hospital and emergency care. Included provider specialties similarly focused on hematology/oncology, radiation oncology, surgery, radiology, and pathology. The full list of included note types and specialties is provided in Supplementary Materials. For each tumor board case, the extracted tumor board date was used as the cutoff date, and only notes documented before that date were retained. Tumor board cases without any eligible prior history notes were excluded. To fit within model context window of approximately 131,072 subtokens, patient histories were capped at 100,000 subtokens to leave room for task instructions and model output. When the full prior history exceeded this limit, we retained the most recent notes within the 100,000-subtoken budget.

### 4.2 Clinical Annotation: Case Summaries, Options, Evaluation

#### Case Summary Processing (Existing Summary/Recommendations Available)

DS1 and DS4 datasets which already had case summaries available required some preprocessing. DS1 case summaries, saved in text format with some repeated and unorganized information, were processed manually by medical oncologists. DS4 was well-structured, but saved as PDFs. Automatic text-conversion and reformatting was performed to extract textual content. Structured fields were retrieved from each protocol text using regular-expression patterns targeting named sections. When Regex extraction failed, an LLM fallback was used to extract the section verbatim from the document text.

#### Case Summary Creation (No Existing Summary Available)

For DS2 and DS5, we first identified tumor board mentions in text. If a date was also mentioned, we created a case time-point for that patient. Medical oncologists were hired to create case summaries and options for DS2, given all prior clinical notes. DS5 dates were recorded from previous meetings and created by medical oncologists after manual review of patient cases.

#### Human Direct Assessment Annotation

**Figure 3.**
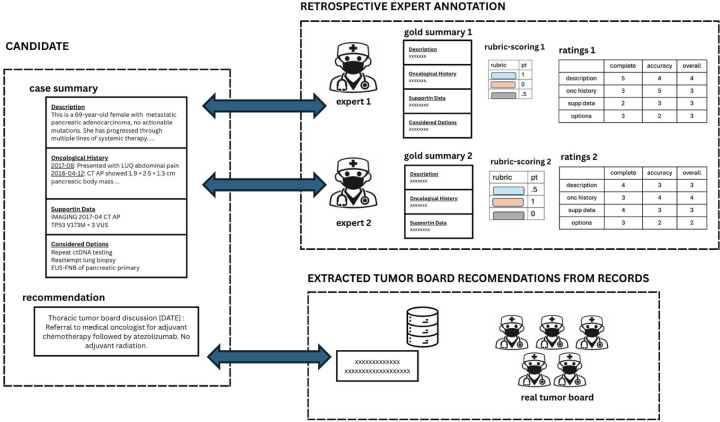
Tumor Board Case Summary, Recommendation, and Evaluation Gold Standard and Annotation Creation. When gold summaries did not exist, they were manually created, along with detailed human direct assessments and rubric scoring for some data subsets. Tumor board recommendations were those from the patient’s actual tumor board discussion.

To obtain human direct assessment annotations, we collected 1–5 Likert scale (5-best) ratings for each output. All case summary outputs from LLM outputs were rated by medical oncologists. The number of ratings per case are shown in Table 3. For DS2 and DS5, tumor board board outcomes were rated for consistency with the final true outcomes by medical annotators and a medical doctor, respectively. DS4 was rated using a Likert scale by a medical oncologist. System outputs are assigned objective assessments (e.g., 1–5); however, they are shown side-by-side, which allows for comparative judgments (e.g., if for the same case, Gemini is rated at 4 and GPT at 5; we may infer GPT was preferred). Moreover unlike previous datasets, human direct assessments are simultaneously accompanied by the manually created references and additional rubric grading annotation (Figure 3). In general, across both datasets, exact F1 agreement is low; however, given a tolerance of 1/5, we see high agreements of >0.8 for case summarization subtasks. For both datasets, the agreement for options generation drops to 0.5 F1 and 0.7 F1, indicating a more challenging task. Detailed breakdowns are available in the Supplementary Materials.

#### Rubric Grading Annotation

DS1 and DS3 were furthermore annotated for rubric points using a gold standard summary. Rubric points were modeled on information extraction frames with an event type trigger/mention (e.g. demographic, stage, treatment_systemic, test_imaging) and its attributes/arguments (e.g. temporal, negation, anatomic location). The full list of event types are specified in the Supplementary Materials. Four system baselines were graded against the gold-standard rubric. Unlike previous rubric grading work^15,16^, added facts (both true facts and hallucinations) were also part of the grading protocol. Rubric criteria were defined based on classic information extraction event schemas with a mention (or event trigger e.g. treatment) and attributes (e.g., negation, temporal, anatomic location). Fact presence (classified for original rubric criteria) was binary (present/not_present). Added facts were assessed for fact accuracy (if true fact) on a binary scale (accurate/not_accurate) and criticality of the missing information (critical/noncritical); if the added fact was a hallucination, harmfulness was classified according to 3 labels (minimal/medium/severe). Expert annotators were instructed to use both the gold standard reference as well as the patient history notes as needed.

We define 3 rubric based scoring methods:

- **rubric-recall:** Similar to traditional school grading, each fact is a single point. One point is given if the mention and attribute presence/not_presence classifications are all correct. Half point if only the mention classification is correct. Zero otherwise. The points are added up and divided only by the facts created from the rubric. Mimics recall by scoring facts appearing from the reference text.
- **rubric-precision:** Scores 1 point for true added facts, 1 point for matching rubric mentions and attributes, 1/2 point for matching mention but mismatched attribute classifications, 0 otherwise. Score is normalized over the number of facts present in the system. This mimics precision by scoring facts appearing from the candidate text.
- **rubric-F1:** Geometric mean of rubric-recall and rubric precision.

We measure grading differences by providing the same set of facts to annotators and calculating the binary classification for mentions and attributes. We found moderately high agreement of 0.841 F1 for mentions and 0.868 F1 for attributes. As added facts (either true because a system can be more detailed than reference), or false (hallucinations) are written in as free text, we compare by taking the absolute differences in number of added facts and hallucinations divided by the mean number of all rubric criteria per case. In both cases, we found less than 10% differences with 8% differences in total added facts and 7% differences in hallucinations out of all rubric criteria. With average rubric facts at 17 items, 33 items counted added facts - this difference may be approximately 3 facts or attributes difference.

### 4.3 System Generations and Automatic Judges

#### Generation Systems: Case Summarization and options generation, Recommendations Prediction

Case Summarization, options generations, and Tumor Board Recommendations Prediction was generated using available LLMs from the DeepSeek, Gemini/Gemma, GPT, and Qwen families shown in Table 4. Each institution had its own restrictions for model availability due to institutional API access constraints or hardware availability - though comparable models were chosen when available. In most experiments a single prompt was used, though DS3 additionally ran an extra filtering and summarization step for each note in the history to decrease input size. Detailed prompts and settings will be released with CODE.

#### TB Eval: LLM-as-judge

LLM-as-Judge has been shown to perform competitively for medical tasks^17^, and in some cases, provide both higher accuracy and more consistency^18^. In this work, we test two settings of LLM-as-Judge: a general score(1–5), as well as 4-axes ratings for completeness, factual-accuracy, relevance, and overall. Example prompts are provided in the Supplementary Materials. The full prompts will be included in the released CODE.

#### Rubric Eval: TBFact and TBFact-RF

Inspired by similar previous work^19–22^, we identify facts from the reference and candidate texts. As a second step, each fact is judged against the other texts as context to see if the fact is entailed by the context. The number of matches is divided by the total number of facts from the reference or candidate, for a recall and precision-like score. While previous work defines facts ambiguously based on LLM generation, here we propose a definition based on domain-motivated frame-based schema developed in collaboration with medical oncologists (same as used in rubric schema).

Specifically, each fact includes a mention and zero or more attributes. Secondly, each fact’s mention and attributes are classified for entailment against the other text with categories: ENTAILED_BY, CONTRADICTED_BY, RELEVANT_BUT_NOT_SUPPORTED and OTHER_UNSUPPORTED. Finally, points are assigned according to the entailment classifications. Specifically, a full point is given for entailed fact mentions with zero or all entailed attributes; a half point is given for entailed fact mentions with at least one unsupported attribute; zero points otherwise. The sum of points is divided by the number of facts. TBFact precision is defined by the number of points divided by the number of facts from the system text, classified for entailment against the reference text. TBFact recall is similarly defined but with facts from the reference text classified against the system text. TBFact F1 is given by the harmonic mean of the two. TBFact-RF (reference-free) instead performs TBFact precision with the entire patient history as context. Further analysis of the metric fidelity can be found in the Supplementary Materials.

## 5 Results

### 5.1 Case Summarization and options generation

Table 5 illustrates the performance of LLM model families for the overall human rating (1–5 Likert scale, 5 is the best) and GPT LLM-as-Judge-Generic performances across datasets. For DS1, DS2, and DS3, both Gemini and GPT models were the strongest competitors, whereas DS4, which used the Gemma and GPT open source equivalents, behaved differently. For the description task, the human ratings for the best system per dataset reached 3.57 for DS1 and 3.37 for DS4, with higher ratings for DS2 at 4.32. Similarly DS1 and DS4 had similar oncological history ratings for Gemini and Qwen respectively at 3.92 and 4.08 overall ratings. The supporting data best ratings were 3.38 for DS1, 4.34 for DS2 and 3.83 for DS4. Options generation scored relatively high for DS2 at 4.06 but low for DS1 at 2.25. Comparing the top-1 ranking per dataset-task, we find that out of the 12 dataset-task combinations, in a modest 7 instances, GPT-as-Judge’s mean ratings were able to correctly identify the best ranked system. More experiments on automatic metric correlations with human ratings are presented in the following sections.

To capture a nuanced picture of quality for realistic deployments, we report the human direct assessments associated with the best performing systems (Table 6). For DS1 and DS2 case summarization tasks, completeness was the major drawback relative to factual accuracy and relevance (helpfulness). However, for options generation, factual accuracy was the weakest axis. This difference is expected, because the previous tasks are extraction or summarization tasks. The options generation task, at times, may take suggestions from the clinical record; however requires generating new ideas.

When measured using the human rubric grading score (manual scoring over individual facts) for description generation (Table 7), GPT scores best for DS1, whereas Gemini scores best for DS3. However, both DS3 systems sit near ceiling at scores of >0.9 out of 1.0. Interestingly, when grading using rubric-recall (akin to grade-school grading) for DS1, the absolute scoring, 40% is much lower than the Likert scale direct assessment which is approximately 80% (4/5). When taking into account just precision of the facts with rubric-precision (# of correct facts/total facts in system), we see a more generous evaluation at 88%; which is closer to either rubric-recall or rubric-F1. These findings suggest that linear grading of individual facts does not necessarily correspond to holistic assessment. We investigate this further in Section 5.3.

Qualitatively, the system outputs with low completeness scores stem from missing input files (due to maximum input constraints). Beyond the objective completeness and accuracy axes, relevance (helpfulness) and final, overall ratings may be heavily influenced by physician and institution preferences. Moreover, when studying gold standard references, especially for DS2, we found large variations in doctor preferred descriptions, oncological history, supporting data, and proposed options. The latter two varied most in the number of items, and the former two varied in the preferred level of detail. Supporting data (e.g. used as in slides for structured output such as graphs or image report excerpts; or as additional other detail) may not always be used by physicians as evidenced in survey results. Finally, options generation likewise varied in detail.

An analysis of experts revealed errors came from both critical inaccuracies and omissions for all sections. There was an expressed additional desire of condensing verbose information for the oncological history section. Finally, options generation main complaints included inaccurate links to publications, omitted key next steps, or inappropriate treatments for current cancers, harmful treatments.

### 5.2 Tumor Board Recommendations Prediction

Table 8 provides the overall scoring of TB recommendations prediction by the best automatic metric and by the human domain experts. Prompts and additional detailed automatic metric results and human evaluation results can be found in the CODE REPOSITORY. As a free text generation task, we find that in general across LLM families, satisfaction with the outputs sat in the 2 of 5 to 3 of 5 range across LLM families, which indicates that the task remains challenging for general-purpose models. Comparing to GPT LLM-as-Judge, we find that though the top system is consistent, the ordering of other systems do not follow the same trend as human evaluations. We explore the automatic evaluation correlation with humans in the next section.

In qualitative analysis, we found that all LLM model families suffered from the same issues across datasets. Mainly, system outputs tended to be unnecessarily verbose and, at times, over-prescribed next steps, and tended to provide more aggressive treatments. For example, a gold recommendation could be for “post operative radiation”, but systems would suggest first obtaining contrast-enhanced CT, then consider re-resection or salvage proton radiotherapy depending on recurrence. One gold recommendation stated that the patient was asymptomatic, the group did not think lymphoma was likely, and the unanimous plan was to follow with MRI scans. Gemma, instead, described refractory DLBCL with new CNS metastases and recommended palliative whole-brain radiation, right hip radiation, a family meeting, and skilled nursing facility discharge. Moreover, models often tried to supply thorough clinical plans instead of predicting the actual tumor board outcomes for required next steps. For example, one gold recommendation was simply to “perform a CT-guided biopsy of this iliac lesion”, but Gemma recommended definitive IMRT to dedifferentiated liposarcoma foci, deferring chemotherapy, continuing pembrolizumab, and longitudinal imaging surveillance.

To provide further insight, medical annotators identified the number of recommendations (defined as all actionable advice), next steps (defined as actionable advice to be done immediately), and next step types (e.g., test, treatment, both) in both gold and system outputs. The results (Table 9) provide interesting trends. Supporting qualitative observations, it is apparent that LLMs tend to over-generate recommendations and next steps - as in both gold standards the average number of recommendations was 1.8–1.9, but in the system outputs, typical recommendations was 3 or more. The tendency for over-advising immediate next steps was less pronounced than the overall total number of recommendations. For both gold-standards, the average number of next steps was a 1.3; both Gemini/Gemma systems provided more, with DeepSeek providing less and Qwen providing more on average. Analyzing the breakdown of next steps, we see that both gold datasets will tend to have a test next step or a treatment next step; in contrast, as evidenced by the higher number of the “test and treatment” category, LLM systems tend to over-provide the immediate next actionable steps.

Finally, as with the case summarization problem, if the underlying clinical note inputs are lacking crucial information - either because of document cut-off or missed critical input notes, the system outputs may try to provide clinically plausible recommendations for the wrong disease context, especially when the input context was too limited. For example, one gold recommendation was “upper endoscopy, endoscopic ultrasound with biopsy”, but Gemma predicted management for stage IV prostate adenocarcinoma, including ADT, radiation to the prostate and lymph nodes, genetic testing, and bone density monitoring. In this case, the input contained only one historical report, which likely did not provide enough longitudinal context for the model to recover the actual tumor board question and recommendation.

### 5.3 Automatic Evaluation Correlation with Human Experts

#### 5.3.1 Correlations of Human Ratings Across All Tasks

Table 10 shows the correlations of LLM-as-Judge-4xes across each dataset-task and across the human direct-assessment axes. Except for the tumor board recommendation task with an average 0.440 average correlation for the overall axis, the most correlations are in the 0.2–0.3 average correlation range across all tasks. DS2 had much lower variations in system performance. We found this due to data skews and specialist differences discussed further in the Section 5.3.2. Among human direct assessment axes averaged across all datasets, completeness was found to be the most correlated at 0.209–0.280; with lower ranges for factual accuracy and relevance.

#### 5.3.2 Case Summarization Description and Recommendations Prediction Correlations

Table 11 provides correlations of automatic metrics against the human overall score for case summarization description sections. TBFact performed either the best or second best across DS1, DS2, and DS4 Case summarization Description generation task. However the same trend was not observed with Recommendations Prediction (Table 12) - for which the LLM-as-Judge scorers performed better.

Across all automatic metrics and tasks, we observed much lower correlations with DS2 with ranges of lower than 10% correlations. On further study, we found two anomalies in the dataset. Firstly, the scores of 4 and 5 accounted for 91% of overall ratings, which is well within inter-rater variability. Secondly, as ratings were paired with rater-written summaries - references could be highly variable. Unlike most work with 1–2 raters covering an entire dataset, we used a large number of experts (13), with different pair combinations per case, leading to inconsistencies in grading. Moreover, we found that ratings and references may not be consistent with self-written references. For example, Table 13 shows two examples of cases. In the first example, the reference is significantly shorter in terms of length and number of facts, but is the system is graded with a 4/5 for completeness. In the second example, the reference is much more detailed than the system output example, but is given 5/5 for all axes. Table 14 provides correlation breakdown by oncologist, as well as their mean relative length differences of their reference vs. system output. These large correlation differences between doctors highlight the challenging problem of measurement when dealing with diverse doctor preference ratings, heterogeneous gold standard references, and inherent flexibility in correctness for summarization tasks.

#### 5.3.3 Correlation of Human Rubric Scoring with Other Metrics

In Table 15, we assess correlation between human holistic direct assessments against granular human rubric scoring for DS1 and DS3. In both datasets, we see the highest correlation of rubric precision (number of true facts/number of system facts) with factual accuracy, 0.517 and 0.797 for DS1 and DS3 respectively, which is consistent with precision/factual-accuracy definitions. Were facts in the gold reference considered the superset of possible facts for the completeness metric, we would expect strong correlations between rubric-recall and completeness. This was the case for DS3 but not for DS1. This is possibly because DS3 included verys strict summary lengths (limited 999 char) allowing gold and system contents to be well aligned without needing to check against patient histories. For DS3 with wilder variations, as with earlier observations, facts from the reference summary need not always be in the candidate description for high completeness scores. If maximizing for overall human direct assessments, we observe that, according to this set of physicians, human rubric precision is much more important than rubric recall.

Table 16 provides automatic metrics correlated against a human rubric-based scoring system. Among all automatic metrics, TBFact-recall had the highest correlations across all rubric-based scores, particularly with rubric-recall. Surprisingly TBFact-precision had negative correlations with all three rubric grading schemas. Analyzing DS1, we found that typical system outputs were longer than the gold standard summary, making an assessment of precision more difficult without access to the entire patient record (as rubric-based scoring allowed access to entire record). On the other hand, for DS3, which featured a strict 999-character length limit (150–200 words) for gold and system summaries, we see that LLM-as-Judge variants performed the best. This difference suggests that more complex metrics (e.g. tbfact-recall, tbfact-F1) are useful for longer contexts while LLM-as-Judge variants do well out-of-the-box for shorter contexts.

#### 5.3.4 TBFact vs TBFact-RF for Case Summarization and TB Recommendations

TBFact (reference-based) and TBFact-RF (reference-free) target the same oncological-fact decomposition but score against different contexts. TBFact entails system facts against a gold reference summary, whereas TBFact-RF entails them against the much larger patient history. The trade-off is that references are expensive to collect, while patient histories are already present in any deployed system. We therefore examine whether removing the reference comes at the cost of human alignment. Table 17 compares the two on DS4 against the human overall rating, and Table 18 shows the per-system absolute scores both metrics assign.

Two observations stand out. First, the reference-based variants track human ratings more closely than the reference-free ones on both tasks. TBFact-F1 yields an average correlation of 0.287 against the human overall rating on case summarization and 0.226 on recommendation prediction, whereas TBFact-RF drops to 0.142 and to a near-zero 0.015 respectively. On recommendation prediction in particular, TBFact-RF correlates with human judgement no better than chance, while TBFact-F1 and TBFact-recall remain informative.

Second, despite the weaker correlation, TBFact-RF systematically assigns higher absolute scores than TBFact-F1, with a 10 to 20 percentage-point gap on case summarization and an even wider gap (up to 0.44 absolute, for Gemma on recommendation prediction) on the shorter recommendation texts. The mechanism is straightforward. The patient history is a much larger context than the reference summary, so the entailment classifier easily finds support for most generated facts even when those facts are clinically peripheral or when the model has missed the points the gold reference emphasizes. The asymmetry is starkest for Gemma, whose recommendation outputs receive TBFact-F1 = 0.166 (low precision-recall agreement with the reference) but TBFact-RF = 0.609 (most generated facts are still grounded somewhere in the chart).

These results indicate that TBFact-RF should not be used as a stand-alone quality proxy. It measures whether generated content is factually anchored in the patient record, but says little about whether the content matches what an oncologist considers the salient summary. Where annotated references are available, the reference-based TBFact-recall is the single most informative fact-based correlate of human ratings on DS4 (avg-corr 0.282 on case summarization and 0.250 on recommendation prediction). TBFact-RF retains value in the no-reference deployment setting, for example as a low-cost factuality gate before a costlier LLM-as-Judge call, but human evaluation or reference-based scoring should be retained for headline quality claims.

## 6 Discussion and Conclusions

In this work, we aggregate cancer cohort datasets from academic medical centers in the United States and Germany, and five datasets for tasks such as tumor board case summarization, options generation, recommendations prediction, and tumor board evaluation. We provide realistic comparisons of current state-of-the-art large language models across four families: DeepSeek, Gemma/Gemini, GPT, and Qwen, with usages of both locally-deployed and API-queried setups, comparing to human expert ratings and automatic ratings. When GPT and Gemini commercial deployments were available, they outperformed other model families. However constrained to open-sourced model settings, DeepSeek and Qwen were more competitive. Across the different tasks, Gemini tended to do better across all settings and tasks.

Unlike summarization, recommendations generation requires access to several pieces of information that is still challenging for general LLM models: (a) outside knowledge, including current oncological protocols and (b) experience and judgment of patient conditions and their likely response to outcomes. The challenge with (a) can be somewhat combated by extending LLM capabilities with RAG systems^23^ – the difficulty would be in the implementation and maintenance. Oncological protocols are constantly evolving and locale-specific with the availability of new drugs, clinical trials, and the cost of local import/export. For access to (b), would require overcoming at least two hurdles: systematic documentation of decision points and ability to train models on large-scale hospital oncological cases.

The results here suggest that metrics work is very much still a challenging task. Although traditionally, automatic metrics rely on a gold standard (often single), this work highlights two critical challenges: (a) how many references are required for metric utilization^24^, (b) how to cope with the noise of variations in rating preferences when system outputs arrive at an already high quality standard. The study of human direct assessments using Likert Scale ratings against human granular rubrics provided a critical enlightening fact. The relationship between piecemeal scoring and overall scoring is non-linear. As many fact-based, rubric-based methods rely on granular grading – it has been shown here that even if LLMs can perfectly generate and grade facts, an extra scoring criteria piece is required for non-linear weightings of facts. Meanwhile, automatic fact-based scoring still faces challenges. We see entailment classification accuracy drops dramatically when two texts’ events are difficult to align (due to summarization compression and a complex number of similar events). Moreover, compute-time was a huge challenge requiring multiple steps: fact extraction, entailment classification, and JSON structuring and verification. Studying TBFact-RF showed entailing over the entire patient record (in some cases 10+ years) dramatically increases compute but did not necessarily help overall correlation.

Finally, our in-depth study on correlations among a variety of physicians reveals an inherent challenge to previous assumptions: objective content differences from a reference is not necessarily indicative of a low human direct assessment score. The correlations of fact-based and LLM-as-Judge approaches vary by dataset and establish a baseline understanding of whether a metric resembles human judgment. Detailed analyses of where metrics and experts disagree^25^ remain a promising direction for future work to examine how automatic LLM-based metrics would shift judgments of model performance. It is also apparent that task tolerance, model ability, and rating clusters are related. For example, for the summarization description generation, there is a huge tolerance for what constitutes “good” vs. “bad” output. On top of this, current LLMs perform well on extractive and abstractive tasks. As a consequence of both, ratings are skewed towards higher ends. On the other hand, for recommendation prediction, the tolerance is lower. Correct recommendations require precise, appropriate tests and treatments given the current condition. Moreover, current models do not perform well, thus human ratings are clustered towards the lower spectrum. To combat such noise in the first case (successful systems, loose grading), requires additional discriminative constraints (e.g., place a word length constraint). For the second case (low performance systems, strict grading), perhaps with better model development evaluation, the next step would be to naturally assess quality based on separate parts of required output (e.g. grade against each required immediate next step).

Key limitations remain in optimizing, evaluating, and deploying tumor board–oriented LLM outputs. Firstly, it is unclear what level of content detail and format best meets diverse physician needs. Secondly, as the technology is not perfect but strongly useful, scalable collection of expert assessments is challenging, as workflow-integrated evaluations risk draft bias, while explicit labeling imposes opportunity costs on clinical care. Integrating tumor board data with EHR systems presents interoperability and regulatory barriers^26^. Finally, enabling large-scale model training on such data requires strong privacy, governance, and patient protection frameworks^27^, particularly given reliance on models developed by private entities. With powerful models generating human-level quality content and judgments at scale, it becomes imperative to critically understand what is being measured and whether the measurements are representative of true task performance. We hope that our work here can educate and inspire further academic research, and encourage health care applications integration and experimentation, ultimately improving patient care quality.

## Supplementary Material

Supplementary Files

This is a list of supplementary files associated with this preprint. Click to download.

- tumorboardnpjDigitalMed20261.pdf

## Figures and Tables

**Table 1.** Treatment Recommendation Task Related Works from Real Clinical Data)

Institution	Cancer Type	Cases	Categorizations	LLMs	Citation
Technical University Munich	Head & Neck	100	Likert Rating of Output	GPT4o and ChatGPT	^5^
Chaim Sheba Medical Center	Breast	10	Concordance of 5 treatment categories	ChatGPT	^8^
LMU University Hospital, Munich	Gastrointe	115	Likert Rating	chatgpt	^11^

**Table 2.** Datasets and Tasks. (Case summarization = Case Summ; Case options generation = Opt Gen; Recommendation Prediction = TB Rec; Tumor Board Summary Rubric Evaluation = Rubric Eval; Overall Evaluation = TB Eval)

Dataset	Cancer Type	Institution	Cases	Case Summ	Opt Gen	TB Rec	Rubric Eval	TB Eval
DS1	Multi	USA	37	✓	✓		✓	✓
DS2	Breast/Lung	Regional-USA	154	✓	✓	✓		✓
DS3	Thoracic	Stanford University	50	✓			✓	✓
DS4	Skin	University of Essen	2182	✓		✓		✓
DS5	Lymphoma/Prostate	University of Washington	83			✓		✓

**Table 3.** Data statistics. Mean lengths are in words. Multiple gold reference may exist per case. DS1 and DS2 evaluated 4 system baselines. Each tumor board case evaluation comprises four ratings (factual accuracy, completeness, relevance/helpfulness, overall) multiplied by the number of sections. Description, Onc. Hist., and Supp. Data denote the description, oncological history, and supporting data sections of the case summary. The DS4 per-section generation counts differ because extracted protocols did not all contain every section.

		Generation					Evaluation		
Dataset	Statistic	Case Summ			Opt Gen	TB Rec	Rubric Eval	TB Eval	
		descript	onchx	supp data				Case Summ	TB Rec
DS1	count	53	53	53	53	–	212	3392	–
N=37	input-length	18186	18186	18186	18186	–	–	–	–
	output-length	161.7	383.4	954.3	408.6	–	–	–	–
DS2	count	302	302	302	302	154	–	4832	154
N=154	input-length	14995	14995	14995	14995	14995	–	–	–
	output-length	185.4	202.6	159.1	609.0	56.8	–	–	–
DS3	count	50	–	–	–	–	100	800	–
N=50	input-length	52520	–	–	–	–	–	–	–
	output-length	36.4	–	–	–	–	–	–	–
DS4	count	1824	1806	2159	–	2182	–	3520	3520
N=variable	input-length	25843	24591	24169	–	23889	–	–	–
	output-length	71.6	159.6	221.9	–	27.4	–	–	–
DS5	count	–	–	–	–	83	–	–	166
N=83	input-length	–	–	–	–	15135	–	–	–
	output-length	–	–	–	–	55.7	–	–	–

**Table 4.** LLM Models Per Institution.

Dataset	DEEPSEEK	GEMMA/GEMINI	GPT	QWEN
DS1/DS2	R1-0528†	Gemini-2.5-pro*	4.1†	qwen-plus-2025-09-11*η*
DS3	–	Gemini-2.5-pro*	4.1†	–
DS4	R1-0528-AWQ	medgemma-27b-it	gpt-oss-120b	Qwen3-Next-80B-A3B-Instruct
DS5	-	Gemma-4-31B	gpt-oss-120b	-

(†=api through Azure AI Foundry, *=api through vertex-ai, *η*=api through alibabacloud.com, *γ*=api through open-ai, local deployments if otherwise not indicated)

**Table 5.** Case Summarization and options generation Overall Performance Across Systems. Exact models are described in the methods section and depended on institutional approval and availability. The GPT-as-Judge column uses the LLM-as-Judge-Generic prompt. Best human ratings per dataset-task are bolded, best automatic rating underlined, ± = standard deviation.

	DeepSeek		Gemma/GEMINI		GPT		Qwen	
section	GPT-as-Judge	human	GPT-as-Judge	human	GPT-as-Judge	human	GPT-as-Judge	human
**DS1**								
descript	2.96 ± 0.83	3.26 ± 1.08	3.19 ± 0.56	**3.57** ± **0.72**	3.23 ± 0.47	**3.57** ± **0.84**	3.08 ± 0.51	3.42 ± 0.77
onchx	2.62 ± 0.97	3.57 ± 1.25	3.11 ± 0.80	**3.92** ± **1.00**	3.09 ± 0.79	3.79 ± 0.91	3.09 ± 0.84	3.75 ± 0.98
supp data	2.47 ± 1.01	3.26 ± 1.08	2.94 ± 0.77	**3.38** ± **0.88**	2.85 ± 0.82	3.32 ± 0.89	2.75 ± 0.83	3.28 ± 0.89
options	2.40 ± 0.88	2.08 ± 0.96	2.62 ± 0.79	2.25 ± 1.02	2.53 ± 0.82	**2.30** ± **1.05**	2.38 ± 0.88	2.19 ± 1.00
**DS2**								
descript	3.59 ± 0.51	4.33 ± 0.68	3.58 ± 0.51	4.32 ± 0.66	3.65 ± 0.50	**4.48** ± **0.63**	3.49 ± 0.53	4.12 ± 0.73
onchx	3.51 ± 0.53	4.35 ± 0.65	3.64 ± 0.53	**4.59** ± **0.57**	3.75 ± 0.45	4.19 ± 0.71	3.66 ± 0.50	4.16 ± 0.72
supp data	3.70 ± 0.56	4.32 ± 0.72	3.97 ± 0.73	4.34 ± 0.75	3.88 ± 0.63	**4.36** ± **0.71**	3.79 ± 0.66	4.27 ± 0.78
options	3.03 ± 0.50	3.81 ± 0.88	3.06 ± 0.51	**4.06** ± **0.84**	3.10 ± 0.44	4.04 ± 0.81	2.90 ± 0.56	3.54 ± 0.91
**DS3**								
descript + onchx	–	–	3.32 ± 0.65	3.96 ± 1.4	3.38 ± 0.64	**4.08** ± **0.87**	–	–
**DS4**								
descript	3.21 ± 1.04	**3.67** ± **0.53**	2.39 ± 1.11	2.93 ± 0.65	3.11 ± 1.05	3.45 ± 0.61	3.09 ± 1.08	3.37 ± 0.61
onchx	3.55 ± 0.88	3.93 ± 0.37	3.35 ± 0.93	3.81 ± 0.57	3.62 ± 0.97	3.69 ± 0.64	3.95 ± 0.81	**4.08** ± **0.57**
supp data	3.45 ± 0.92	**3.83** ± **0.36**	2.96 ± 0.97	3.43 ± 0.67	3.52 ± 1.00	3.72 ± 0.59	3.49 ± 0.96	**3.83** ± **0.61**

**Table 6.** Case Summarization and options generation Performance For Best Systems. The GPT-as-Judge column uses the LLM-as-Judge-4axes prompt, ± = standard deviation.

	Human Direct Assessment							
	Completeness		Factual Accuracy		Relevance		Overall	
section(best sys)	GPT-as-Judge	human	GPT-as-Judge	human	GPT-as-Judge	human	GPT-as-Judge	human
**DS1(GEMINI)**								
descript	4.21 ± 0.69	3.17 ± 0.58	4.08 ± 0.68	4.32 ± 0.98	4.68 ± 0.64	3.91 ± 0.86	3.94 ± 0.66	3.57 ± 0.72
onchx	4.11 ± 0.85	3.94 ± 0.97	3.81 ± 0.90	4.47 ± 1.03	4.45 ± 0.93	4.36 ± 0.98	3.75 ± 0.90	3.92 ± 1.00
supp data	2.96 ± 0.71	3.09 ± 0.86	3.85 ± 0.77	4.43 ± 0.97	3.68 ± 0.89	3.83 ± 1.09	2.91 ± 0.69	3.38 ± 0.88
options	3.40 ± 0.66	2.43 ± 1.08	4.34 ± 0.59	2.11 ± 1.58	3.72 ± 0.77	2.47 ± 1.14	3.32 ± 0.70	2.25 ± 1.02
**DS2 (GEMINI)**								
descript	4.31 ± 0.51	4.32 ± 0.72	4.51 ± 0.57	4.64 ± 0.72	4.94 ± 0.25	4.79 ± 0.51	4.25 ± 0.54	4.32 ± 0.66
onchx	4.43 ± 0.58	4.65 ± 0.62	4.58 ± 0.55	4.79 ± 0.53	4.91 ± 0.35	4.73 ± 0.51	4.29 ± 0.58	4.59 ± 0.57
supp data	3.70 ± 0.78	4.21 ± 0.92	4.48 ± 0.76	4.79 ± 0.57	4.56 ± 0.69	4.73 ± 0.60	3.68 ± 0.79	4.34 ± 0.75
options	3.89 ± 0.91	4.35 ± 0.74	4.52 ± 0.61	4.16 ± 1.04	4.08 ± 0.87	4.22 ± 0.86	3.83 ± 0.87	4.06 ± 0.84
**DS3(GPT)**								
descript + onchx	4.72 ± 0.57	4.56 ± 0.86	4.74 ± 0.56	4.66 ± 0.89	4.9 ± 0.36	4.0 ± 0.73	4.62 ± 0.60	4.08 ± 0.87
**DS4 (Qwen)**								
descript	3.34 ± 1.06	3.49 ± 0.61	2.80 ± 1.05	3.45 ± 0.74	3.52 ± 1.01	3.40 ± 0.65	2.92 ± 0.85	3.37 ± 0.61
onchx	4.48 ± 0.76	4.31 ± 0.56	4.20 ± 0.97	4.00 ± 0.54	4.36 ± 0.69	4.25 ± 0.63	4.04 ± 0.73	4.08 ± 0.57
supp data	4.04 ± 0.88	4.04 ± 0.52	3.62 ± 1.26	3.77 ± 0.63	4.06 ± 0.87	3.96 ± 0.67	3.56 ± 0.88	3.83 ± 0.61

**Table 7.** Human Rubric Scoring for Description Generation (scale 0 to 1, 1 best, best system bolded, ± = standard deviation). For DS3, the summary combines the description and oncological-history sections.

	Human Rubric Grading		
system	rubric-recall	rubric-precision	rubric-F1
**DS1**			
DeepSeek	0.439 ± 0.04	0.796 ± 0.03	0.534 ± 0.04
Gemini	0.437 ± 0.05	0.855 ± 0.04	0.544 ± 0.05
GPT	**0.461** ± **0.08**	**0.879** ± **0.03**	**0.576** ± **0.05**
Qwen	0.449 ± 0.09	0.844 ± 0.05	0.557 ± 0.09
**DS3**			
Gemini	**0.962** ± **0.09**	**0.985** ± **0.04**	**0.971** ± **0.06**
GPT	0.921 ± 0.16	0.981 ± 0.07	0.944 ± 0.12

**Table 8.** Tumor Board Recommendations Prediction Scores (Best human ratings per dataset-task bolded, best automatic rating underlined, ±=standard deviations)

dataset	system	GPT-as-Judge	human score
**DS2**	DeepSeek	2.63 ± 0.89	1.97 ± 0.80
	Gemma/GEMINI	2.66 ± 0.95	**2.27** ± **1.04**
	GPT	2.56 ± 0.89	2.18 ± 0.92
	Qwen	2.28 ± 0.95	1.90 ± 0.79
**DS4**	DeepSeek	2.27 ± 0.98	**3.67** ± **0.55**
	Gemma/GEMINI	1.64 ± 0.84	2.82 ± 0.74
	GPT	1.93 ± 0.89	3.39 ± 0.60
	Qwen	2.18 ± 0.99	3.26 ± 0.66
**DS5**	Gemma	2.33 ± 1.17	**2.19** ± **0.97**
	GPT	2.34 ± 1.09	2.17 ± 1.05

**Table 9.** Tumor Board Recommendations Statistics (±=standard deviations)

Recommendation	DS2					DS5		
	Gold	System				Gold	System	
		DeepSeek	Gemini	GPT	Qwen		Gemma	GPT
Number Recommendations	1.8 ± 0.9	4.0 ± 1	2.3 ± 0.9	3.2 ± 1.1	4.7 ± 1.3	1.9 ± 1.1	3.3 ± 1.1	5.1 ± 1.5
Number Next Steps	1.3 ± 0.6	1.0 ± 1.0	1.5 ± 0.7	1.9 ± 0.9	2.4 ± 1.1	1.3 ± 0.8	2.4 ± 1.1	3.3 ± 1.5
Next Steps								
test	32%	42%	44%	39%	36%	18%	25%	17%
treatment	48%	30%	44%	34%	34%	51%	24%	18%
test and treatment	8%	30%	11%	26%	29%	12%	41%	60%
other	11%	0%	0%	0%	1%	19%	10%	5%

**Table 10.** Correlations for **Human Direct Assessments** against **GPT LLM-as-Judge-4axes** (ALL represents the average across dataset-tasks) τ=kendall’s tau, *r*=pearson, *ρ*=spearman, avg-corr=mean of τ, *r*, *ρ*)

	Human Direct Assessments															
	Completeness				Factual Accuracy				Relevance				Overall			
tasks	*τ*	*r*	*ρ*	avg-corr	*τ*	*r*	*ρ*	avg-corr	*τ*	*r*	*ρ*	avg-corr	*τ*	*r*	*ρ*	avg-corr
**DS1**																
descript	0.242	0.433	0.259	0.311	0.157	0.375	0.179	0.237	0.207	0.476	0.231	0.305	0.199	0.413	0.219	0.277
onchx	0.214	0.417	0.239	0.290	0.211	0.365	0.234	0.270	0.134	0.287	0.150	0.190	0.195	0.427	0.229	0.283
supp data	0.277	0.387	0.314	0.326	0.170	0.275	0.192	0.212	0.256	0.403	0.299	0.319	0.283	0.403	0.320	0.335
options	0.306	0.386	0.352	0.348	0.045	0.061	0.052	0.053	0.276	0.361	0.319	0.319	0.281	0.333	0.323	0.312
**DS2**																
descript	0.067	0.054	0.069	0.063	0.073	0.080	0.077	0.077	0.046	0.035	0.047	0.043	0.040	0.047	0.042	0.043
onchx	0.070	0.073	0.072	0.071	−0.097	−0.095	−0.100	−0.097	−0.073	−0.071	−0.078	−0.074	−0.163	−0.163	−0.171	−0.166
supp data	0.192	0.231	0.212	0.212	0.043	0.052	0.045	0.046	0.075	0.087	0.079	0.081	0.129	0.164	0.142	0.145
options	0.098	0.133	0.109	0.113	−0.010	−0.007	−0.011	−0.010	0.093	0.107	0.106	0.102	0.014	0.026	0.015	0.018
tb rec	–	–	–	–	–	–	–	–	–	–	–	–	0.475	0.523	0.547	0.515
**DS3**																
descript + onchx	0.310	0.465	0.325	0.366	0.337	0.506	0.354	0.399	0.043	0.006	0.047	0.032	0.228	0.476	0.256	0.320
**DS4**																
descript	0.288	0.338	0.352	0.326	0.200	0.246	0.250	0.232	0.207	0.286	0.266	0.253	0.234	0.302	0.293	0.276
onchx	0.212	0.323	0.263	0.266	0.007	0.050	0.009	0.022	0.074	0.131	0.093	0.099	0.163	0.226	0.198	0.195
supp data	0.241	0.308	0.294	0.281	0.178	0.176	0.223	0.192	0.146	0.187	0.178	0.170	0.188	0.230	0.233	0.217
tb rec	0.329	0.400	0.407	0.379	0.279	0.338	0.358	0.325	0.255	0.301	0.320	0.292	0.258	0.329	0.314	0.300
**DS5**																
tb rec	–	–	–	–	–	–	–	–	–	–	–	–	0.652	0.723	0.733	0.703
**ALL**																
descript	0.227	0.323	0.251	0.267	0.192	0.302	0.215	0.236	0.126	0.201	0.148	0.158	0.175	0.31	0.203	0.229
onchx	0.165	0.271	0.191	0.209	0.04	0.107	0.048	0.065	0.045	0.116	0.055	0.072	0.065	0.163	0.085	0.104
supp	0.25	0.316	0.293	0.286	0.148	0.187	0.178	0.171	0.171	0.241	0.206	0.206	0.209	0.275	0.247	0.243
options	0.236	0.311	0.283	0.277	0.08	0.111	0.102	0.098	0.175	0.225	0.21	0.203	0.179	0.229	0.213	0.206
tb rec	–	–	–	–	–	–	–	–	–	–	–	–	0.462	0.525	0.531	0.506

**Table 11.** Correlations with **Human Overall Metric** for **Case Summarization - Description** (τ=kendall’s tau, *r*=pearson, *ρ*=spearman, avg-corr=mean of τ, *r*, *ρ*, best correlations bolded, 2nd best underlined)

	DS1				DS2				DS3				DS4			
metric	*τ*	*r*	*ρ*	avg-corr	*τ*	*r*	*ρ*	avg-corr	*τ*	*r*	*ρ*	avg-corr	*τ*	*r*	*ρ*	avg-corr
BLEU	0.025	0.128	0.036	0.063	0.058	0.058	0.074	0.063	0.180	0.111	0.243	0.178	0.154	0.173	0.216	0.181
ROUGE	0.094	0.424	0.121	0.213	0.055	0.046	0.07	0.057	0.204	0.267	0.258	0.243	0.193	0.273	0.268	0.245
LLM-as-Judge	0.125	0.34	0.136	0.201	0.068	0.088	0.071	**0.076**	0.197	0.389	0.219	0.268	0.170	0.213	0.208	0.197
LLM-4xes-overall	0.199	0.413	0.219	0.277	0.04	0.047	0.042	0.043	0.228	0.476	0.256	**0.320**	0.257	0.333	0.319	**0.303**
TBFact-F1	0.254	0.458	0.318	**0.343**	0.048	0.088	0.06	0.065	0.062	0.145	0.077	0.095	0.218	0.342	0.301	0.287
TBFact-precision	0.022	−0.204	0.025	−0.052	0.044	0.074	0.055	0.058	0.001	0.063	0.007	0.024	0.140	0.179	0.194	0.171
TBFact-recall	0.231	0.413	0.29	0.311	0.029	0.041	0.037	0.036	0.136	0.258	0.174	0.189	0.216	0.334	0.298	0.282

**Table 12.** Correlations with **Human Overall Metric** for **Recommendation Prediction** (τ=kendall’s tau, *r*=pearson, *ρ*=spearman, avg-corr=mean of τ, *r*, *ρ*, best correlations bolded, 2nd best underlined)

	DS2				DS4				DS5			
metric	*τ*	*r*	*ρ*	avg-corr	*τ*	*r*	*ρ*	avg-corr	*τ*	*r*	*ρ*	avg-corr
BLEU	0.211	0.208	0.28	0.233	0.004	0.063	0.004	0.024	0.117	0.278	0.161	0.185
ROUGE	0.203	0.258	0.266	0.242	0.145	0.183	0.201	0.176	0.194	0.332	0.269	0.265
LLM-as-Judge	0.44	0.489	0.488	0.472	0.277	0.345	0.338	**0.320**	0.564	0.661	0.670	0.632
LLM-4xes-overall	0.475	0.523	0.547	**0.515**	0.258	0.329	0.314	0.300	0.587	0.669	0.691	**0.649**
TBFact	0.306	0.382	0.364	0.351	0.181	0.246	0.251	0.226	0.419	0.555	0.511	0.495
TBFact-precision	0.275	0.336	0.331	0.314	0.020	0.021	0.029	0.023	0.402	0.475	0.491	0.456
TBFact-recall	0.322	0.353	0.402	0.359	0.198	0.285	0.267	0.250	0.437	0.623	0.543	0.535

**Table 13.** DS2 Example Reference-Candidate-Rating Triples. (Top) Although the reference is shorter, the candidate is rated relatively high for completeness. (Bottom) Although the reference is more detailed than the candidate, the expert still rated full marks for the system output.

**AUTHOR_ID:** Oncologist-05
**REFERENCE:** 64 year old man with past medical history of active tobacco use for 50 years, CAD, diabetes, and stroke who presents with biopsy-proven lung squamous cell cancer from a right upper lobe biopsy. CT brain demonstrated left parietal lobe mass. Molecular testing, neurosurgery, and radiation oncology consults pending. PD-L1 negative. We are presenting the case to discuss further workup and treatment options.
**GEMINI:** This is a 64-year-old man with newly diagnosed Stage IVA (cT1b, cN1, cM1b) poorly differentiated squamous cell carcinoma of the left upper lobe. Biomarkers are p40 positive, rare TTF-1 positive, and PD-L1 negative. He has a significant smoking history and comorbidities including diabetes, hypertension, and severe obstructive lung disease (FEV1 40%). He presented with respiratory symptoms and headaches; workup including a PET scan on [DATE: 23494] revealed a 2 cm FDG-avid left upper lobe mass with ipsilateral hilar/mediastinal adenopathy. A recent CT brain for worsening headaches revealed a left parietal lobe lesion concerning for metastasis. He is presented to the tumor board to discuss the management of oligometastatic disease. Questions include the optimal treatment and sequencing for his brain metastasis (SRS vs. surgery) and thoracic disease, and whether he is a candidate for definitive chemoradiation given his poor pulmonary function.
Completeness: 4, Factual-Accuracy: 5, Relevance: 5, Overall: 4
**AUTHOR_ID:** Oncologist-09
**REFERENCE:** 78 year-old, ex-smoker with 15 pack years, quit [DATE: 15960], retired dentist who presented with cough and haemoptysis. He developed persistent haemoptysis in [DATE: 28659] (coughing up blood-streaked mucus). Chest CT showed a RML infiltrate/mass. He was treated with 10 days of Levaquin but continued to cough up small amounts of bright red blood in the morning. Repeat chest CT on [DATE: 28756] showed enlarging 2.5 × 6.4 cm right lower lung lesion (compared to 2.1 × 5.7cm on [DATE: 28733] causing mass effect on adjacent airway. The lesion was noted to be PET/CT positive. No endobronchial lesion noted on bronchoscopy. Right lung biopsy on [DATE: 28755] confirmed mucinous adenocarcinoma. Patient underwent Right VAT, right thoracotomy, right lower lobectomy, mediastinal lymph node dissection on [DATE: 28784]. Final staging: pT3N2 stage: IIIA PMH of atrial fibrillation on apixaban 5mg BD, prostate cancer s/p prostatectomy, mild CAD, HTN, and hyperlipidaemia. For tumour board discussion to review pathology result and consideration of adjuvnat treatment
**GEMINI:** This is a 78-year-old man with newly diagnosed, surgically resected pathologic Stage IIIA (pT3N2) invasive mucinous adenocarcinoma of the right lower lobe. His comorbidities include atrial fibrillation on apixaban, hypertension, and a history of prostate cancer status post prostatectomy. He is a former smoker with a good performance status. He presented with hemoptysis, and imaging revealed a progressively enlarging RLL mass. He underwent a right lower lobectomy with mediastinal lymph node dissection on [DATE: 28784], confirming a completely resected 8 cm pT3N2 tumor. The patient is presented to discuss recommendations for adjuvant therapy. What is the optimal adjuvant treatment strategy, considering his age, histology, and pT3N2 staging?
Completeness: 5, Factual-Accuracy: 5, Relevance: 5, Overall: 5

**Table 14.** DS2 Correlations by Doctor of **Human Overall Metric** against **LLM-as-Judge-4axes-Overall** for **Case Summarization - Description** (±=standard deviations, nan occurs when the doctor only used one rating, τ=kendall’s tau, *r*=pearson, *ρ*=spearman, avg-corr=mean of τ, *r*, *ρ*,)

expert	cases	%len-diff	*τ*	*r*	*ρ*	avg-corr
oncologist-01	204	20 ± 27	0.181	0.177	0.189	0.182
oncologist-02	36	18 ± 27	0.150	0.153	0.154	0.152
oncologist-03	48	36± 18	0.392	0.384	0.410	0.395
oncologist-04	28	6± 31	0.071	0.016	0.078	0.055
oncologist-05	24	26 ± 22	nan	nan	nan	nan
oncologist-06	48	50± 16	0.131	0.153	0.133	0.139
oncologist-07	104	57 ± 14	−0.000	0.038	−0.000	0.013
oncologist-08	8	49 ± 15	0.420	0.421	0.436	0.426
oncologist-09	124	−35 ± 51	0.099	0.099	0.102	0.100
oncologist-10	168	17 ± 21	0.183	0.198	0.198	0.193
oncologist-11	16	−22 ± 22	0.062	0.064	0.065	0.064
oncologist-12	392	−25 ± 48	−0.082	−0.075	−0.085	−0.081
oncologist-13	8	45 ± 14	0.000	−0.000	0.000	−0.000

**Table 15.** Correlations with **Human Direct Assessment** with **Human Rubric Evaluation** for **Case Summarization - Description**. GPT versions of LLM based metrics are shown in this table. (τ=kendall’s tau, *r*=pearson, *ρ*=spearman, avg-corr=mean of τ, *r*, *ρ*, best correlation with direct assessments bolded)

	Human Rubric Grading											
	rubric-recall				rubric-precision				rubric-F1			
metric	*τ*	*r*	*ρ*	avg-corr	*τ*	*r*	*ρ*	avg-corr	*τ*	*r*	*ρ*	avg-corr
**DS1**												
completeness	0.323	0.466	0.399	0.396	0.311	0.657	0.384	**0.450**	0.329	0.502	0.408	0.413
factual-accuracy	0.184	0.314	0.239	0.246	0.399	0.649	0.501	**0.517**	0.198	0.371	0.260	0.276
relevance	0.250	0.407	0.318	0.325	0.179	0.628	0.227	**0.345**	0.249	0.463	0.318	0.343
overall	0.279	0.429	0.350	0.353	0.280	0.649	0.347	**0.425**	0.286	0.482	0.360	0.376
**DS3**												
completeness	0.623	0.710	0.675	**0.669**	0.429	0.624	0.455	0.502	0.606	0.742	0.656	0.668
factual-accuracy	0.441	0.476	0.481	0.466	0.714	0.783	0.754	**0.750**	0.551	0.601	0.609	0.587
relevance	0.008	0.113	0.011	0.044	0.073	0.113	0.080	**0.089**	0.037	0.140	0.044	0.074
overall	0.306	0.411	0.346	0.355	0.513	0.670	0.557	**0.580**	0.389	0.515	0.444	0.449

**Table 16.** Correlations of **Automatic Metrics** with **Human Rubric Evaluation** for **Case Summarization - Description**. GPT versions of LLM based metrics are shown in this table. (τ=kendall’s tau, *r*=pearson, *ρ*=spearman, avg-corr=mean of τ, *r*, *ρ*, best average correlation bolded, 2nd best underlined)

	Human Rubric Grading											
	rubric-recall				rubric-precision				rubric-F1			
metric	*τ*	*r*	*ρ*	avg-corr	*τ*	*r*	*ρ*	avg-corr	*τ*	*r*	*ρ*	avg-corr
**DS1**												
bleu	0.236	0.336	0.335	0.303	0.068	0.237	0.103	0.136	0.234	0.379	0.336	0.316
rouge	0.206	0.350	0.275	0.277	0.048	0.636	0.069	0.251	0.200	0.423	0.271	0.298
LLMAsJudgeGeneric	0.011	0.115	0.018	0.048	0.111	0.548	0.139	0.266	0.008	0.159	0.014	0.060
LLMAsJudge4Axes-completeness	0.183	0.310	0.230	0.241	0.160	0.613	0.203	0.325	0.188	0.346	0.236	0.257
LLMAsJudge4Axes-factual-accuracy	0.217	0.333	0.272	0.274	0.187	0.537	0.243	0.322	0.217	0.350	0.274	0.280
LLMAsJudge4Axes-relevance	0.130	0.272	0.161	0.188	0.090	0.620	0.116	0.275	0.129	0.309	0.160	0.199
LLMAsJudge4Axes-overall	0.192	0.304	0.241	0.246	0.175	0.549	0.228	0.317	0.193	0.324	0.244	0.254
TBFact-precision	−0.119	−0.267	−0.177	−0.188	−0.099	−0.476	−0.143	−0.239	−0.122	−0.316	−0.181	−0.206
TBFact-recall	0.577	0.780	0.764	**0.707**	0.254	0.488	0.361	**0.368**	0.571	0.775	0.763	**0.703**
TBFact-F1	0.294	0.453	0.420	0.389	0.133	0.487	0.192	0.271	0.290	0.474	0.414	0.392
**DS3**												
bleu	0.102	0.107	0.136	0.115	0.084	0.100	0.102	0.095	0.120	0.113	0.157	0.130
rougeL	0.174	0.218	0.222	0.205	0.153	0.250	0.190	0.198	0.186	0.241	0.239	0.222
LLM-as-Judge	0.291	0.405	0.316	0.337	0.267	0.474	0.283	0.341	0.313	0.457	0.344	0.371
LLM-as-Judge-4xes-completeness	0.370	0.433	0.405	**0.403**	0.380	0.502	0.398	0.426	0.406	0.487	0.448	**0.447**
LLM-as-Judge-4xes-factual-accuracy	0.217	0.285	0.233	0.245	0.353	0.533	0.368	0.418	0.244	0.371	0.266	0.294
LLM-as-Judge-4xes-relevance	0.254	0.268	0.273	0.265	0.397	0.483	0.412	**0.431**	0.328	0.343	0.359	0.343
LLM-as-Judge-4xes-overall	0.325	0.384	0.356	0.355	0.382	0.507	0.402	0.430	0.362	0.449	0.400	0.404
TBFact-precision	0.048	0.093	0.065	0.069	0.071	0.113	0.090	0.091	0.079	0.115	0.102	0.099
TBFact-recall	0.213	0.268	0.268	0.250	0.152	0.199	0.186	0.179	0.233	0.269	0.297	0.267
TBFact-F1	0.082	0.151	0.106	0.113	0.132	0.155	0.165	0.151	0.123	0.171	0.158	0.151

**Table 17.** Correlations of TBFact variants and TBFact-RF with the **Human Overall** rating on DS4. Case Summarization aggregates the summary and oncological-history sections (n=500 pairs); Recommendation Prediction is one row per case-candidates (n=250 pairs). (τ=Kendall’s tau, *r*=Pearson, *ρ*=Spearman, avg-corr=mean of the three, best average correlation bolded, 2nd best underlined)

	DS4 Case Summarization (Description)				DS4 Recommendation Prediction			
metric	*τ*	*r*	*ρ*	avg-corr	*τ*	*r*	*ρ*	avg-corr
TBFact-F1	0.218	0.342	0.301	**0.287**	0.181	0.246	0.251	0.226
TBFact-precision	0.140	0.179	0.194	0.171	0.020	0.021	0.029	0.023
TBFact-recall	0.216	0.334	0.298	0.282	0.198	0.285	0.267	**0.250**
TBFact-RF	0.128	0.120	0.179	0.142	0.018	0.002	0.027	0.015

**Table 18.** Mean DS4 scores assigned by TBFact (F1, reference-based) versus TBFact-RF (grounding, reference-free) per system. Case Summarization pools summary and oncological-history sections; Recommendation Prediction is the recommendation section. TBFact-RF is uniformly higher and the gap is largest on Recommendation Prediction for Gemma (bold).

	DeepSeek		Gemma		GPT		Qwen	
task / metric	TBFact-F1	TBFact-RF	TBFact-F1	TBFact-RF	TBFact-F1	TBFact-RF	TBFact-F1	TBFact-RF
Case Summarization	0.609	0.710	0.606	0.806	0.621	0.792	0.639	0.761
Recommendation Pred.	0.288	0.414	0.166	**0.609**	0.240	0.374	0.284	0.487

## Data Availability

The data used in this study cannot be shared publicly due to patient privacy considerations. However, we invite collaboration opportunities with the corresponding author for each institution, subject to institutional approval. We will release our guidelines for rubric creation and rubric grading to share with the community.

## Footnotes

1 Categories included: surgical treatment (ST), endocrine treatment (ET), systemic treatment or chemotherapy (CT), radiation therapy (RT) and genetic testing (GT)

## Code availability

Our prompts and code are available at (GITHUB REPO TO BE RELEASED ON PUBLICATION).
